# Acquired spontaneous periodic hypothermia and pancytopenia secondary to hypothalamic compression from a large skull base meningioma

**DOI:** 10.1007/s10072-026-09059-4

**Published:** 2026-05-07

**Authors:** Sagar Jolly, Bilal Ali, Rishabh Jain, Shashank Paliwal, Sreerashmi Sasikumar, Delatre Lolo

**Affiliations:** 1 Department of Anesthesiology, New York Medical College, Metropolitan Hospital Center, 1901 1st Ave, New York, NY 10029 USA; 2 Department of Interventional Radiology, Fortis Escorts Heart Institute, Okhla, New Delhi India; 3 Department of Anaesthesiology, Critical Care and Pain Medicine, All India Institute of Medical Sciences, Bathinda, Punjab India; 4 Department of Internal Medicine, New York Medical College, Metropolitan Hospital Center, New York, USA

**Keywords:** Spontaneous periodic hypothermia, Hypothalamic dysfunction, Neuroendocrine dysfunction, Skull base meningioma, Pancytopenia

## Abstract

A 69-year-old woman with a recurrent skull-base meningioma developed recurrent hypothermia during rehabilitation, initially misattributed to infection. Episodes followed a circadian pattern and were associated with delirium, pancytopenia, and signs of central endocrine dysfunction. MRI showed a large meningioma compressing the hypothalamus with an intact corpus callosum. Infection and adrenal insufficiency were excluded. Hypothermia resolved with external warming, and pancytopenia normalized spontaneously. Subclinical hypothyroidism and central hypogonadism were noted, consistent with hypothalamic-pituitary axis involvement. These findings supported a diagnosis of acquired spontaneous periodic hypothermia (SPH) due to hypothalamic compression. This case illustrates that early recognition of SPH in patients with skull-base tumors can prevent misdiagnosis and guide appropriate supportive care and monitoring.

## Case presentation

**Fig. 1:**
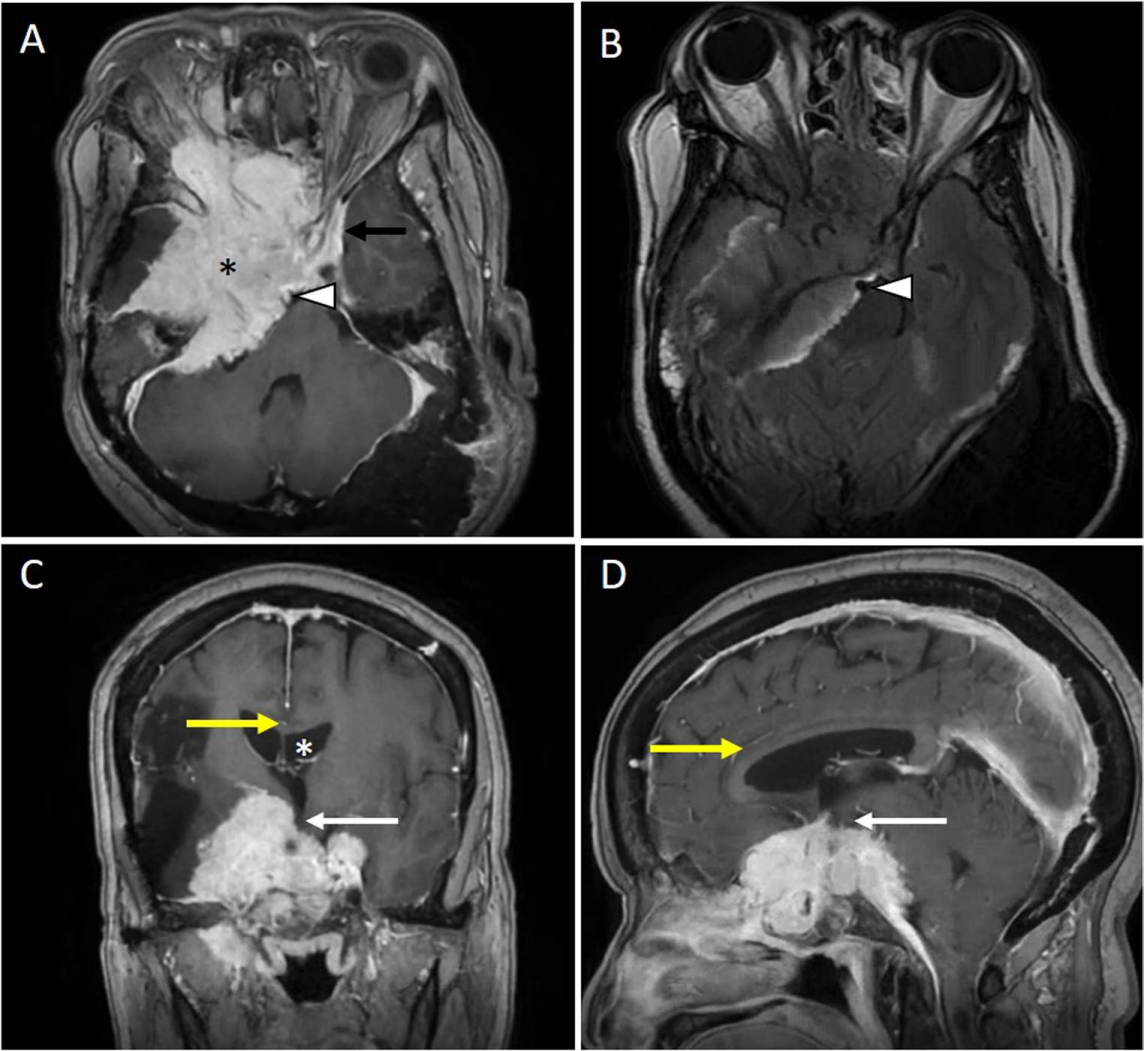
Post-contrast enhanced brain MRI shows a large skull-base lesion arising from the right greater wing of the sphenoid (Panel **A**, *) and extending into the right orbit, nasopharynx, cavernous sinus (normal left cavernous sinus is marked by a black arrow in Panel **A**). The mass is encasing the basilar artery (Panel **A** & **B**, white arrowheads). Superiorly, it extends to the floor of the third ventricle, involving the hypothalamus (Panel **C** & **D**, white arrows). The corpus callosum is away from the lesion and remains uninvolved (Panel **C** & **D**, yellow arrow). Associated hydrocephalus is noted (White * in Panel **C**)

A 69-year-old woman with a history of craniotomy in 2009 for a large skull-base meningioma, complicated by a right middle cerebral artery territory stroke, was transferred to our acute rehabilitation facility following a complex neurosurgical admission for resection of a recurrent, invasive skull-base tumor. MRI revealed two distinct lesions. The first was a recurrent meningioma arising from the right sphenoid wing with extensive invasion into the cavernous sinus, sellar and suprasellar regions, clivus, nasopharynx, and right orbital apex. The lesion encased the right optic nerve, internal carotid artery, and anterior cerebral artery, with mass effect on the third ventricle and hypothalamus (Fig. 1). The second lesion was an intraosseous meningioma of the left posterior occipital bone with transosseous expansion crowding the posterior fossa.

The patient underwent staged neurosurgical interventions, including endovascular embolization of tumor feeders, suboccipital craniectomy with C1 laminectomy to decompress a coexisting Chiari malformation and C2-T4 syrinx, and subsequent left occipital craniectomy for resection of the intraosseous lesion. A right VP shunt was placed to address symptomatic hydrocephalus and right temporal horn entrapment.

During the subacute postoperative phase following the VP shunt procedure, she developed recurrent episodes of profound hypothermia, prompting external warming with thermal blankets. Initial endocrine evaluation showed ACTH 13.0 pg/mL, TSH 2.3 µIU/mL, and morning cortisol of 11 mcg/dL, all within reference limits. She was diagnosed with a urinary tract infection (Klebsiella pneumoniae) and treated with ceftriaxone. Hypothermia episodes during that admission, with a temperature nadir of 33.1 °C, were attributed to infection and resolved with antibiotics.

Upon transfer to the rehabilitation unit, she again developed profound hypothermia (27.3 °C) and was admitted to the inpatient service. She appeared drowsy, lethargic, and disoriented to time, with generalized weakness greater on the left side and no shivering despite hypothermia. Vital signs revealed stable hemodynamics (HR 72 bpm, BP 122/67 mmHg), and an oral temperature of 33.4 °C. She experienced acute delirium, which resolved by day five following rewarming and non-pharmacologic interventions. Lab results revealed pancytopenia (WBC count 2.89 × 10³/µL, HGB 7.3 g/dL, and PLT 123 × 10³/µL). Markers of hemolysis were present, including elevated LDH (408 U/L), low haptoglobin (24 mg/dL), and compensatory reticulocytosis (3.01%). Peripheral smear findings supported a reactive rather than marrow-suppressive process. Infectious workup, including procalcitonin (0.06 ng/mL), respiratory viral-panel, urinalysis, and cultures were negative.

**Fig. 2:**
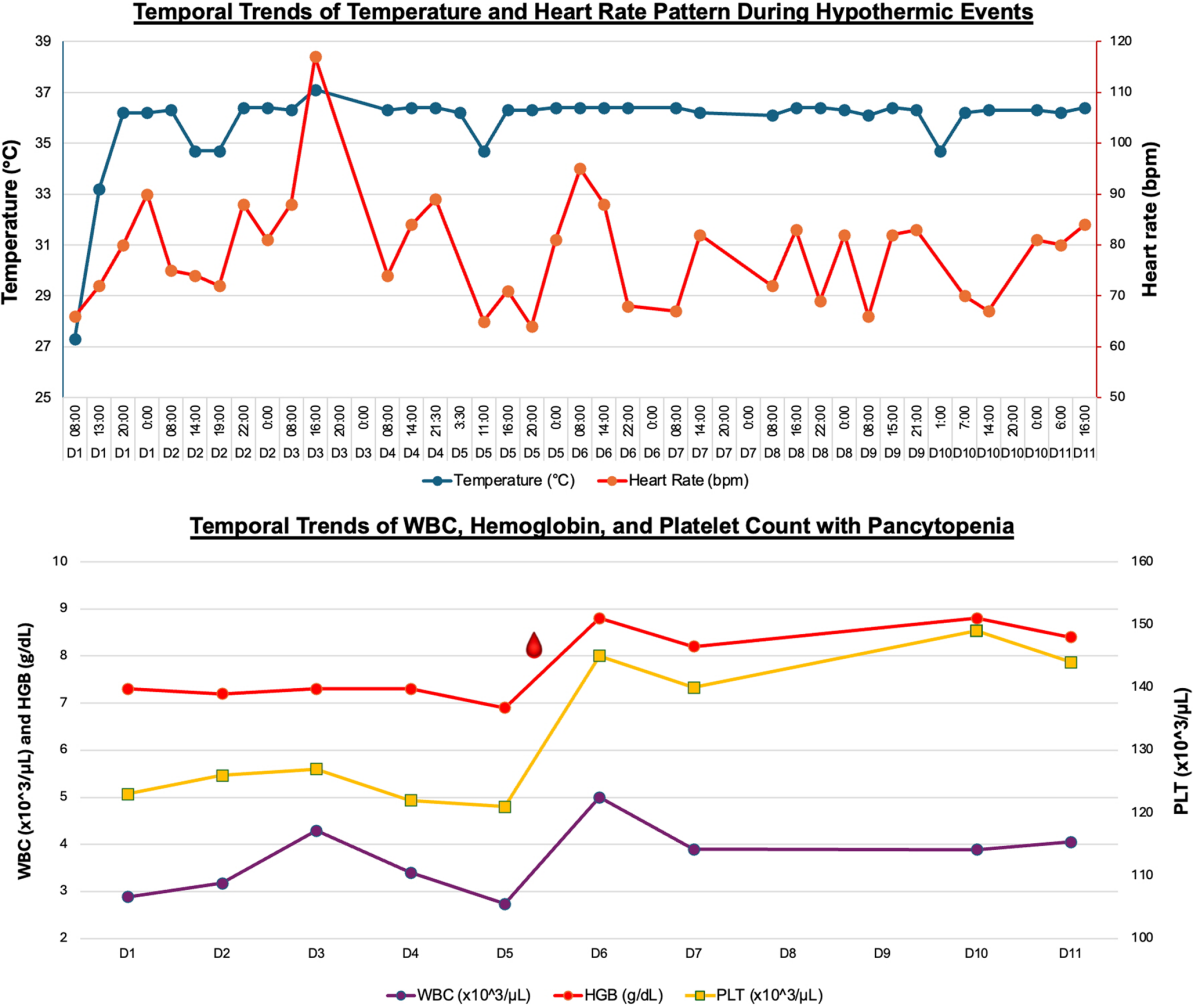
Temporal trends in vital signs and hematologic parameters during hypothermia episodes and pancytopenia (1 unit PRBC transfusion given at Day 5)

Trend analysis of vital signs over 11 days revealed a circadian pattern to hypothermic episodes, with temperature nadirs during early morning hours accompanied by decreased HR (Fig. 2). All episodes resolved with external warming interventions. Endocrine studies revealed mildly elevated TSH (6.79 µIU/mL) with normal free T4 (1.1 ng/dL), suggestive of subclinical hypothyroidism, and suppressed gonadotropins (FSH < 0.3, LH < 0.3 mIU/mL), indicating central hypogonadism. ACTH levels (22.3 and later 34.8 pg/mL) were normal, and a cosyntropin stimulation test confirmed intact adrenal function. Collectively, these findings were consistent with central thermoregulatory dysfunction secondary to hypothalamic compression.

To understand how hypothalamic compression produced this constellation of findings, it is useful to review the central thermoregulatory circuitry. The hypothalamus serves as the principal thermoregulatory center, integrating peripheral thermal inputs and coordinating autonomic and behavioral responses to maintain body temperature within a narrow physiological range. Afferent thermal signals ascend via the spinothalamic and trigeminothalamic tracts to the preoptic area (POA) of the hypothalamus, known as the body’s thermostat. The POA coordinates downstream autonomic pathways controlling vasoconstriction, shivering, and thermogenesis. Recent studies have highlighted the integrated role of the POA, dorsomedial hypothalamus, and paraventricular nucleus in circadian thermoregulation and acute thermoregulatory responses [1, 2].

**Fig. 3:**
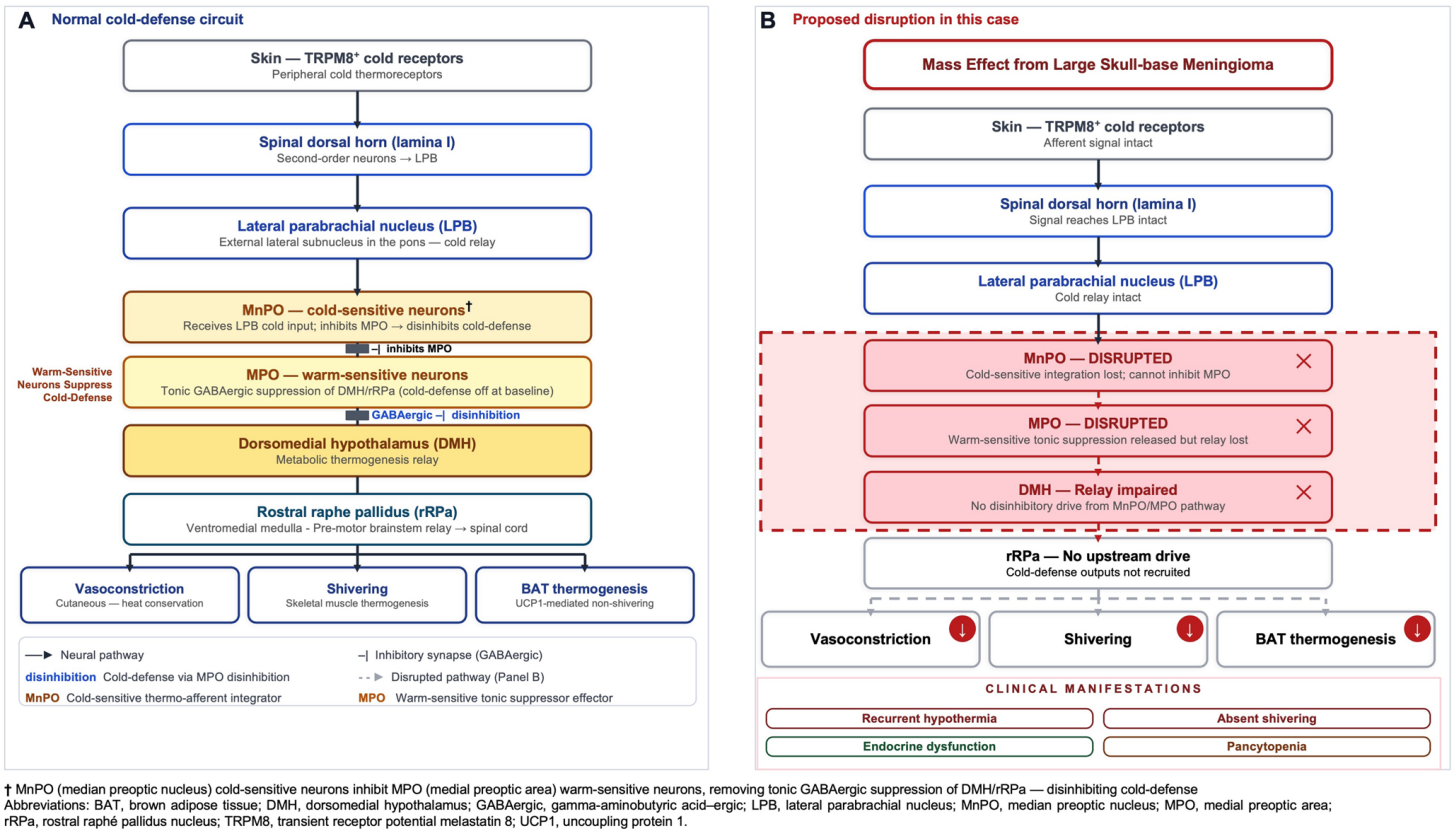
Hypothalamic cold-defense circuit and proposed mechanism of acquired spontaneous periodic hypothermia secondary to skull-base meningioma. Panel **A**: Cold signals ascend from TRPM8⁺ cutaneous receptors via the spinal dorsal horn and LPB to the MnPO, which inhibits warm-sensitive MPO neurons, releasing their tonic GABAergic suppression of the DMH and rRPa, activating vasoconstriction, shivering, and BAT thermogenesis. Panel** B**: Mass effect from a skull-base meningioma disrupts the MnPO–MPO–DMH axis abolishing cold-defense disinhibition and producing the clinical triad of recurrent hypothermia, absent shivering, and endocrine dysfunction, with peripheral cold afferent signaling remaining intact. †Circuit adapted from Morrison and Nakamura [1] and Tansey and Johnson et al. [2]

Under physiologic conditions, cold signals detected by cutaneous TRPM8⁺ thermoreceptors ascend via the spinal dorsal horn and lateral parabrachial nucleus (LPB) to the median preoptic nucleus (MnPO), the primary thermoafferent integrator of the hypothalamus (Fig. 3, Panel A). Warm-sensitive neurons of the adjacent medial preoptic area (MPO) tonically suppress cold-defense outputs at baseline through GABAergic projections to the dorsomedial hypothalamus (DMH) and rostral raphe pallidus nucleus (rRPa) [1, 2]. Cold activation of MnPO neurons inhibits these MPO neurons, releasing their suppressive hold on the DMH and rRPa, a process termed disinhibition, which thereby activates cutaneous vasoconstriction, shivering thermogenesis, and brown adipose tissue-mediated nonshivering thermogenesis.

In this case, MRI showed a large skull-base meningioma with mass effect on the hypothalamus and the floor of the third ventricle (Fig. 1), compressing the hypothalamic nuclei central to cold-defense thermoregulation, impairing both thermal sensing and heat-generation pathways, resulting in recurrent hypothermia. Compressive injury to the anterior and tuberal hypothalamus in this patient disrupted the MnPO–MPO–DMH axis (Fig. 3, Panel B), abolishing cold-defense disinhibition and silencing all three thermoeffector outputs, leading to profound hypothermia, complete absence of shivering despite a temperature nadir of 27.3 °C, and inability to achieve spontaneous rewarming. Preserved peripheral cold sensation favored a central, rather than peripheral, thermoregulatory defect; this interpretation was further supported by the systematic exclusion of infectious, metabolic, and adrenal causes of hypothermia.

The patient’s clinical course was consistent with acquired spontaneous periodic hypothermia (SPH), characterized by a circadian pattern of recurrent hypothermic episodes, absence of shivering or sweating, subjective sensation of feeling hot at near-normal body temperatures, altered mental status, and no evidence of active infection [3]. Unlike classical Shapiro syndrome, typically associated with corpus callosum agenesis, acquired SPH has been reported secondary to structural hypothalamic lesions [3–6]. In this case, the corpus callosum remained intact, supporting the diagnosis of an acquired rather than congenital SPH.

Acquired SPH has been linked to various intracranial pathologies affecting hypothalamic function, including lipomas, craniopharyngiomas, gliomas, and other compressive lesions [3, 4, 7–9]. Our patient’s hypothermic episodes demonstrated a clear circadian pattern, with temperature nadirs occurring during the early morning when core body temperature naturally reaches its physiologic low point [10]. This exaggerated circadian fluctuation suggests partial, not complete, hypothalamic dysfunction, with preserved but dysregulated central thermoregulation.

Resolution of the hypothermic episodes required active external rewarming, as the patient lacked endogenous heat-generating responses, most notably the complete absence of shivering despite a temperature nadir of 27.3 °C. This observation supported a centrally mediated thermoregulatory failure rather than a systemic cause, with external warming functioning not simply as supportive care but as a physiologic substitute for impaired autonomic cold-defense mechanisms. Management was intentionally conservative; episodes were hemodynamically tolerated and reliably reversed with rewarming. The clinical context weighed against empiric pharmacologic therapy: pre-existing atrial fibrillation on anticoagulation raised proarrhythmic concern with clomipramine, and concurrent delirium precluded agents with anticholinergic or sedating properties such as cyproheptadine. Although clonidine, cyproheptadine, and clomipramine have been described in spontaneous periodic hypothermia, the evidence is limited to case reports and small series, responses are inconsistent, and no standardized treatment strategy exists [3, 5]. The evidence base is even more limited in secondary forms caused by structural hypothalamic lesions. Should episodes recur with greater frequency, severity, or refractoriness to warming, an individualized pharmacologic trial could be considered, although the evidence supporting such therapy remains anecdotal.

In addition to thermoregulatory dysfunction, the patient developed transient pancytopenia during episodes of profound hypothermia. Laboratory findings, including elevated LDH, low haptoglobin, and reticulocytosis, were consistent with acute hemolysis. Such hematologic abnormalities in SPH are linked to red blood cell membrane instability, hepatic and splenic sequestration, and transient suppression of bone marrow output under hypothermic stress [11–14]. The spontaneous normalization of blood counts following rewarming in our patient supports a reactive process rather than primary marrow failure.

Endocrine evaluation revealed selective hypothalamic-pituitary axis dysfunction. While hypothalamic-pituitary-adrenal (HPA) axis function was preserved, indicated by normal cortisol levels and a normal cosyntropin response, the patient exhibited central hypogonadism and subclinical hypothyroidism. This pattern aligns with reports of hypothalamic lesions producing variable effects on different endocrine pathways, reflecting the regional susceptibility of specific hypothalamic nuclei to mass effect [15].

This case illustrates how mass effect from a skull-base meningioma can produce clinically significant hypothalamic compression, resulting in acquired SPH, endocrine dysfunction, and reversible hematologic abnormalities. Recognition of these patterns is essential for timely diagnosis and supportive care.

## Conclusion

This is a rare presentation of an extensive skull-base meningioma manifesting with acquired SPH, secondary to hypothalamic compression, resulting in recurrent, circadian-pattern hypothermia, endocrine dysfunction, and transient pancytopenia. The presence of hypothermia without shivering, particularly when following a circadian rhythm and in the absence of infection, should prompt consideration of central thermoregulatory disruption in patients with hypothalamic-adjacent tumors. Continuous trend-based monitoring of vital signs is essential, as spot measurements may miss patterns, leading to the misattribution of hypothermia to infection, adrenal insufficiency, or hypothyroidism. External active warming remains the cornerstone of supportive management in central hypothermia, as endogenous heat-generating mechanisms are often impaired.

Additionally, reversible pancytopenia during hypothermic episodes highlights the need to differentiate reactive hematologic changes secondary to hemolysis, peripheral sequestration, and margination of WBCs due to hypothermia rather than primary bone marrow pathology, potentially avoiding unnecessary invasive investigations. This case underscores the value of a multidisciplinary approach in identifying and managing atypical presentations of central hypothermia.

## Data Availability

Not Applicable.
